# Functional study of *CYP90A1* and *ALDH3F1* gene obtained by transcriptome sequencing analysis of *Brassica napus* seedlings treated with brassinolide

**DOI:** 10.3389/fpls.2022.1040511

**Published:** 2022-11-03

**Authors:** Qingqin Gan, Mingbao Luan, Maolong Hu, Zhongsong Liu, Zhenqian Zhang

**Affiliations:** ^1^ College of Agriculture, Hunan Agricultural University, Changsha, China; ^2^ Institute of Bast Fiber Crops, Chinese Academy of Agricultural Sciences/Key Laboratory of Stem-Fiber Biomass and Engineering Microbiology, Ministry of Agriculture, Changsha, China; ^3^ Institute of Industrial Crops, Jiangsu Academy of Agricultural Science, Nanjing, China

**Keywords:** Sclerotinia, herbicide, *Brassica napus*, brassinolide, *ALDH3F1*, *CYP90A1*, overexpression

## Abstract

Sclerotinia disease and weeds of *Brassica napus* greatly reduce crop yields. However, brassinolides can improve the resistance of plants to sclerotinia diseases and herbicides. In this study, we investigated the effects of brassinolide on the occurrence, physiological indices, yield, and gene expression of Fanming No. 1 seeds under sclerotinia and glufosinate stress. The results showed that soaking of the seeds in 0.015% brassinolide for 6 h reduced the incidence of sclerotinia by 10%. Additionally, in response to glufosinate stress at the seedling stage, the enzyme activities of catalase and superoxide dismutase increased by 9.6 and 19.0 U/gFW/min, respectively, and the soluble sugar content increased by 9.4 mg/g, increasing the stress resistance of plants and yield by 2.4%. *LHCB1*, *fabF*, *psbW*, *CYP90A1*, *ALDH3F1*, *ACOX1*, *petF*, and *ACSL* were screened by transcriptome analysis. *ALDH3F1* and *CYP90A1* were identified as key genes. Following glufosinate treatment, transgenic plants overexpressing ALDH3F1 and CYP90A1 were found to be resistant to glufosinate, and the expression levels of the *ALDH3F1* and *CYP90A1* were 1.03–2.37-fold as high as those in the control. The expression level of *ATG3*, which is an antibacterial gene related to sclerotinia disease, in transgenic plants was 2.40–2.37-fold as high as that in the control. Our results indicate that these two key genes promote plant resistance to sclerotinia and glufosinate. Our study provides a foundation for further studies on the molecular mechanisms of rapeseed resistance breeding and selection of new resistant varieties.

## 1 Introduction

Rapeseed is the second-largest oilseed crop (Asaduzzaman et al., 2014). As one of the fastest growing global sources of edible oilseeds, rapeseed is among the few species with the potential for meeting the growing edible oil requirements of many countries in Asia, Africa, and America (Sharma et al., 2012).

Weed infestation during the rapeseed seedling stage is a major factor reducing yield (Krato and Petersen, 2012). Although chemical herbicides can effectively and easily control weeds, pesticide residues are extremely harmful to the growth of crops, particularly rapeseed (Gondo et al., 2021). Sclerotiorum is one of the main pathogens that causes serious stem rot disease in *Brassica napus L.* (Bolton et al., 2006). The serious stem rot disease in Brassica napus L. that is caused by sclerotiorum results in a yield loss of 10-70% in rapeseed cultivation (Del Río et al., 2007; Derbyshire and Denton-Giles, 2016). Disease control in rapeseed relies mainly on partially resistant lines which is resist sclerotiorum (Wang et al., 2018). The molecular mechanisms underlying the response of the rapeseed plant to this pathogen are poorly understood (Zhang et al., 2022).

Brassinolides improve plant adaptation to biotic and abiotic stresses, such as those caused by heavy metals, pesticides, herbicides, and organic pollutants (Rajewska et al., 2016; Yuan et al., 2017; Xia et al., 2018). Cytochrome P450 participates in the biosynthesis of endogenous lipophilic compounds, such as fatty acids, brassinolides, and gibberellins, and it enables the oxidative detoxification of many herbicides (Mary, 2010). *CYP90A1* engages in the most important step in brassinolide biosynthesis (Szekeres et al., 1996). In wheat (*Triticum aestivum*), a series of P450s mediate the N-demethylation and cyclo-methylhydroxylation of phenylurea herbicides such as primosulfuron (Frear DS); in soybean, *CYP71A10* N-demethylates a range of phenylurea herbicides and ring-methyl hydroxylates chlortoluron (Siminszky et al., 1999).

*ALDH* belongs to a family of NAD(P)+-dependent enzymes with broad substrate specificity and catalyzes the oxidation of various toxic aldehydes to carboxylic acids. Transgenic *Arabidopsis* plants overexpressing *Ath-ALDH3* show improved tolerance to dehydration, NaCl, heavy metals, and methyl viologen (Sunkar et al., 2003). In contrast, deletion of *ALDH* affects photosynthesis in plants. The protective effects of *ALDH3F1* are required to maintain membrane fluidity and support leaf gas exchange and photosynthesis (Zhao, J. Y. et al., 2017).

We previously showed that ATG3 can improve rapeseed resistance to *Sclerotinia sclerotiorum* (Wang et al., 2019). Treatment of *Brassica napus* seeds with brassinolides can improve resistance to herbicides (glufosinate-ammonium) at the seedling stage. Transcriptome analysis revealed that *ALDH3F1* and *CYP90A1* were involved in these effects. Overexpression of *ALDH3F1* and *CYP90A1* improved the tolerance of rapeseed to glufosinate-ammonium and enhanced *ATG3* expression. In this study, we investigated the underlying mechanisms influencing stress resistance, which may help accelerate molecular breeding of herbicide-resistant rapeseed plants.

## 2 Materials and methods

### 2.1 Plant material

Fanming No. 1 was cultivated in an experimental field at the Yunyuan Experimental Base of Hunan Agricultural University (Changsha, Hunan Province) at a planting density of 30 × 30 cm. Experiments were performed in triplicate according to planned regular regional experiments for Fanming No. 1. The plot area was 10 m^2^, and the plants were planted in rows.

At the seedling stage, the control was soaked in water and sprayed with 88.8% ammonium glyphosate (dilution ratio 400×) and 200 g/L glufosinate (dilution ratio 400×). Young leaf tissue samples were collected on days 7, 10, and 13 after treatment, with five samples collected per treatment. The samples were divided into two parts: one part was immediately frozen at −80°C and the other was used to extract RNA. We performed physicochemical analysis of the frozen samples. For the samples evaluating using quantitative reverse transcription polymerase chain reaction (qRT-PCR), the first sampling time was October 28, 2021 and the last sampling time was November 3, 2021. The first sampling time for samples used to analyze physicochemical traits was November 3, 2021 and the last sampling time was April 27, 2022. Mature *B. napus* plants were harvested on May 3, 2022, and agronomic traits were determined.

### 2.2 Measurement items and methods

#### 2.2.1 Effect of brassinolide on Fanming No. 1

##### 2.2.1.1 Treatment methods

Fanming No. 1 seeds (n = 100) were soaked in water or 0.15% and 0.015% brassinolide solutions for 0, 2, 4, 6, 8, and 10 h. The germination rate was measured to determine the optimum concentration and soaking time.

##### 2.2.1.2 Effects of brassinolide on the physiology, biochemistry, yield, and quality of Fanming No. 1

Leaf samples were taken from the third-to-last leaf of the plant to measure superoxide dismutase (SOD) (Gao, 2006), peroxidase (Bestwick et al., 1998), and catalase activities (Hayat et al., 2016). The contents of malondialdehyde (Vos et al., 1991), protein (Bradford, 1976), total sugar (Dubois et al., 1951), and total chlorophyll (Anis et al., 2020) were determined using a U 8000 spectrophotometer (METASH, Shanghai, China). All experiments included three biological replicates, each containing five plants.

To determine the agronomic traits, the field growth of Fanming No. 1 was investigated in January 2022. The agronomic traits, yield, and quality of rapeseed at maturity were investigated in May 2022. All experiments were performed in five biological replicates.

#### 2.2.2 Field disease investigation

At 35 days after pollination of Fanming No. 1, the presence of disease (sclerotiorum) was evaluated on all plants of each line, as described by Wang et al. (2019).

#### 2.2.3 Key differential gene screening

##### 2.2.3.1 RNA extraction from plant samples

The third-to-last leaf at the 5–6 leaf stage of Fanming No. 1 was collected, and more than three leaves from the brassinolide soaking treatment and control groups were collected. One sample was frozen in liquid nitrogen (−80°C). The TransZol Up Plus RNA Kit reagent was used for RNA extraction (Beijing TransGen Biotech Co., Ltd., Beijing, China). RNA quality was evaluated using a Nanodrop2000 (Thermo Fisher Scientific, Waltham, MA, USA) and 2100 Bioanalyzer (Agilent Technologies, Santa Clara, CA, USA).

##### 2.2.3.2 Transcriptome sequencing

Transcriptome sequencing analysis was performed by Nanjing Pesennuo Gene Technology Ltd. (Nanjing, China).

##### 2.2.3.3 Sequencing result analysis

We focused on metabolic pathways related to herbicide resistance, such as photosynthesis (Song et al., 2020), pyruvate metabolism (Leslie and Baucom, 2014), aromatic amino acid synthesis (Zhao N. et al., 2017), and screened key differential genes combined with functional annotations provided by NCBI.

#### 2.2.4 Functional gene screening

The third to last leaves of each treatment sample were collected at different stages from more than three plants. One sample was cryopreserved in liquid nitrogen at −80°C and RNA was extracted using a TransZol Up Plus RNA Kit (Beijing TransGen Biotech Co., Ltd., Beijing, China). RNA quality was detected using a Nanodrop 2000 (Thermo Fisher Scientific) and 2100 Bioanalyzer (Agilent Technologies). cDNA was synthesized using approximately 0.5 μg of RNA and PrimeScript RT Master Mix (Aidlab Biotechnologies Co., Ltd., Changsha, China). qRT-PCR was performed for each sample using a Bio-Rad CFX96 Touch Detection System (Hercules, CA, USA) and SYBR Green PCR Master Mix (Aidlab Biotechnologies Co., Ltd.). Primers for the qRT-PCR experiments (**Table 1**) were designed using NCBI, and eight glufosinate resistance-related genes were analyzed. We used a qRT-PCR system and procedure developed using the SYBR Green PCR Master Mix Kit (Aidlab Biotechnologies Co., Ltd.). After PCR amplification, quantitative changes in each gene were analyzed using the delta Ct method (Livak and Schmittgen, 2001).

**Table 1:** Primer sequences.

Gene name	Primer name	Sequence (5’-3’)
*BnActin*	BnActin -F	CGTTGGTGGAGTTGCACTTG
	BnActin -R	AGCACGTTACGGGATTGGTT
*psbW*	psbW -F	CTGGTCTTTCTCTCTGAACAT
	psbW -R	AACAACAAGAAACCAGAAGATCA
*petF*	petF -F	CCTTCCAAAAGCCACTGCCC
	petF -R	AGAGACTCGCACTGTAGCCA
*LHCB1*	LHCB1 -F	CTCCATGTTTGGATTCTTTGTA
	LHCB1 -R	ACATCACATTCAAGATTTAACAA
*fabF*	fabF -F	ATCTCTACCGCTTGTGCTACTT
	fabF -R	TGTGACAATGCCCTACAAGC
*ACSL*	ACSL -F	AATGGATAGTTGCTGGGATG
	ACSL -R	GAGAGAGTTTCCCAGCTTTA
*ALDH3F1*	ALDH3F1 -F	TCTTGTCAGAAACATCGTCAG
	ALDH3F1 -R	GAGGGTATCGAGCTTCCAGAT
*ACOX1*	ACOX1 -F	CCTTTTATCTCGTCGTCTCC
	ACOX1 -R	CGATCTCTAGATGACAGCAC
*CYP90A1*	CYP90A1 -F	CTCATGCTTGATATTGACCG
	CYP90A1 -R	AGAAGAGAGGGAGAGGTATTG
*ALDH3F1*	K-ALDH3F1 -F	CTTCTGAACAGAGTCGAGGG
	K-ALDH3F1 -R	TGTGTGTGTTTCTCTTATCGCT
*ALDH3F1*	A-XbaI-F	gagaacacgggggactctagaATGGAAGCCATGAAGGAGACTG
	A-BamAI-R	gcccttgctcaccatggatccTCTTTTAAGACCGAGCATTAAGAGG
*CYP90A1*	K-CYP90A1 -F	CCACTCTCCCCCTCTCCATT
	K-CYP90A1 -R	CAAGTAGCGGATAAGCCACCA
*CYP90A1*	C-XbaI-F	gagaacacgggggactctagaATGGCTTTCTCCTTCTCCTCCA
	C-BamAI-R	gcccttgctcaccatggatccAGTAGCGGATAAGCCACCATCA
*ATG 3*	ATG 3-F	TCGGCGTTCAAGGAGAAG
	ATG 3-R	TGCCAGGGTCACCAGATT

#### 2.2.5 Gene function verification

##### 2.2.5.1 Gene cloning and construction of overexpression vector

RNA was extracted using the TransZol Up Plus RNA kit. First-strand cDNA was synthesized from 1 μg of RNA using a Maxima H Minus First-Strand cDNA Synthesis Mix Kit (Thermo Fisher Scientific) according to the manufacturer’s instructions. Synthetic cDNA was used to amplify the coding sequences of *ALDH3F1* and *CYP90A1* with the primers listed in **Table 1**. The amplified product was detected using 1% agarose gel electrophoresis and 0.1% GelRed nucleic acid gel staining, inserted into the PEASY vector (TransGen Biotech), and verified by sequencing. The *ALDH3F1* and *CYP90A1* sequences were inserted into the XbaI and BamHI restriction sites of the vector PBI121 to construct recombinant plasmids. The *ALDH3F1*-PBI121 and *CYP90A1*-PBI121 recombinant plasmids were introduced into *Agrobacterium tumefaciens* strain LBA4404 to overexpress *Brassica napus* (Zhongshuang 11).

##### 2.2.5.2 Agrobacterium-mediated transformation of Zhongshuang 11

Rape hypocotyls were obtained and transformed as described by Eva et al. (2011). The explants were subcultured in the medium every 15 days. Selected green calli were transferred to induction medium. After two months, the selected shoots were transferred to the rooting medium. The medium formulation was prepared as described by Baskar et al. (2016).

##### 2.2.5.3 Gene function verification

To verify *ALDH3F1* and *CYP90A1* genes function, glufosinate (2000× solution) was applied to the unfolded leaves of each rape seedling as described by Cui et al. (2016) with some modifications. Leaf performance was observed on day 7, after which the leaves were collected for qRT-PCR analysis.

### 2.3 Statistical analysis

Microsoft Excel 2010 software (Redmond, WA, USA) was used to sort data. All experimental data are presented as the average of three independent biological replicates. Data were analyzed by one-way analysis of variance using SPSS software (version 22.0; SPSS, Inc., Chicago, IL, USA). Different letters indicate significantly different means within the same group (*p* < 0.05, Duncan’s multiple range test).

## 3 Results

### 3.1 Optimum conditions for brassinolide processing

Seeds from *B. napus* (Fanming No. 1) were soaked in brassinolide solution or water indoors (approximately 25°C). The effect of the soaking time on the germination rate is shown in **Table 2**. Soaking in 0.015% brassinolide for 6 h showed the better than other treatments.

**Table 2:** The soaking time test of brassinolide.

Soaking time (h)		0	2	4	6	8	10
Germination rate (%) Germination percentage	Water	100	100	100	94	88	82
	0.15% Brassinolide	100	100	96	92	86	78
	0.015% Brassinolide	100	100	100	100	90	86

**Figure 1:**
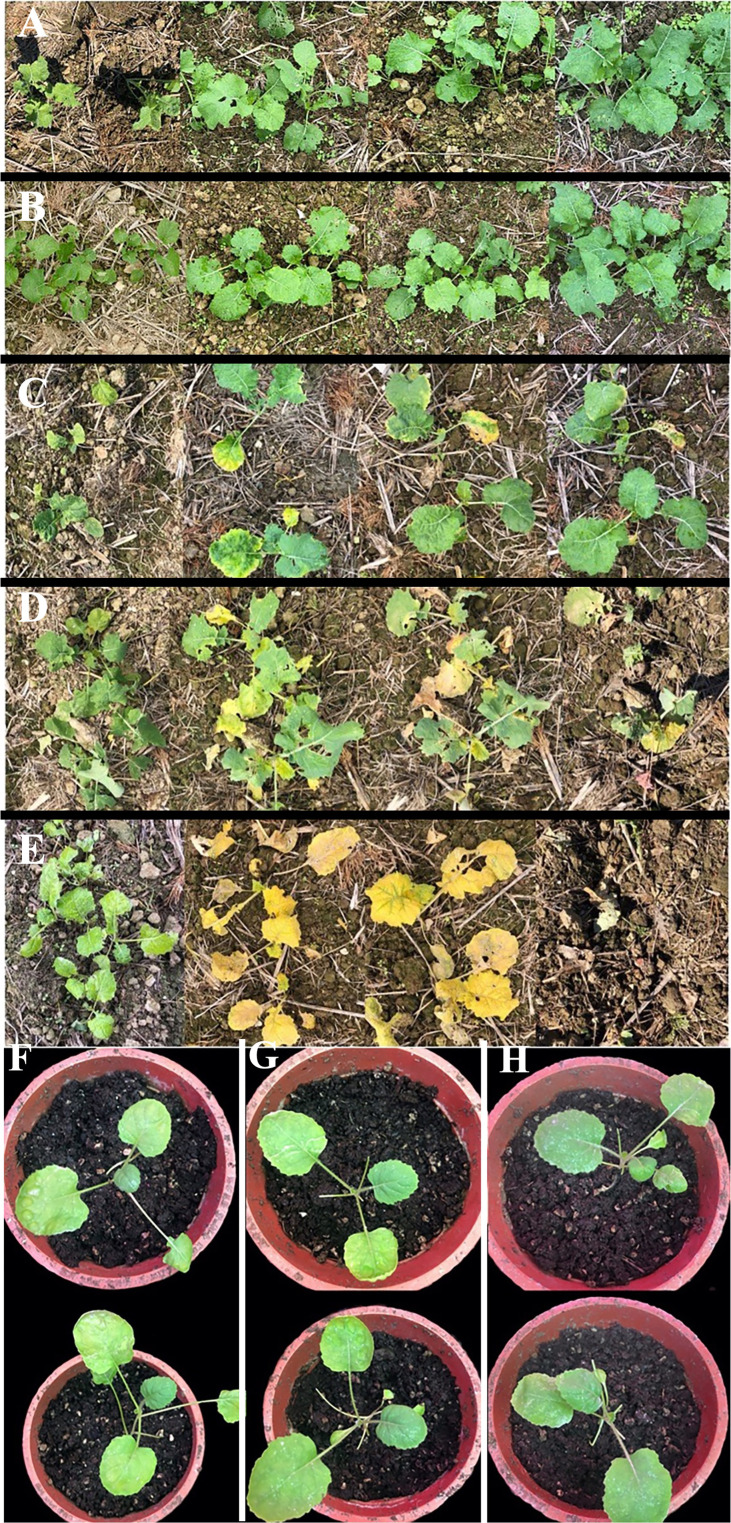
*Brassica napus* under different treatments. **(A)** water treatment, **(B)** 0.015% brassinolide treatment, **(C)** 0.015% brassinolide treatment and glufosinate-ammonium dilution ratio of 400× treatment, **(D)** 0.015% brassinolide treatment and glyphosate dilution ratio of 400× treatment, and **(E)** water treatment and dilution ratio of 400× glufosinate-ammonium treatment. **(A–E)** Performance of Fanming No. 1 under different treatments at 0, 7, 10, and 13 days after herbicide treatment. **(F–H)** *Brassica napus*, *ALDH3F1* transgenic *B napus*, and *CYP90A1* transgenic *B napus*, respectively; day 7 after smearing the leaves with 2000X glufosinate solution.

Herbicide spraying was performed at the 4–5 leaf stage (early November) of Fanming No. 1. The plants showed similar growth under different treatments, namely, A (blank control) and B (brassinolide treatment). The growth of rapeseed in group C (brassinolide treatment and herbicide glufosinate-ammonium treatment) was strongly affected, with the leaves turning yellow and continuing to grow after 13 days (**Figure 1**). Group D plants (brassinolide and herbicide glyphosate treatments) withered on day 13 and died on the day after glyphosate treatment. In group E (herbicide glufosinate-ammonium treatment), after glufosinate treatment, the whole plant turned yellow on day 7 and died on this day. These results demonstrate that soaking of Fanming No. 1 seeds in brassinolide improved the resistance of seedlings to glufosinate.

### 3.2 Effect of brassinolide treatment on Fanming No. 1

#### 3.2.1 Analysis of physiological and biochemical indices of Fanming No. 1

**Figure 2:**
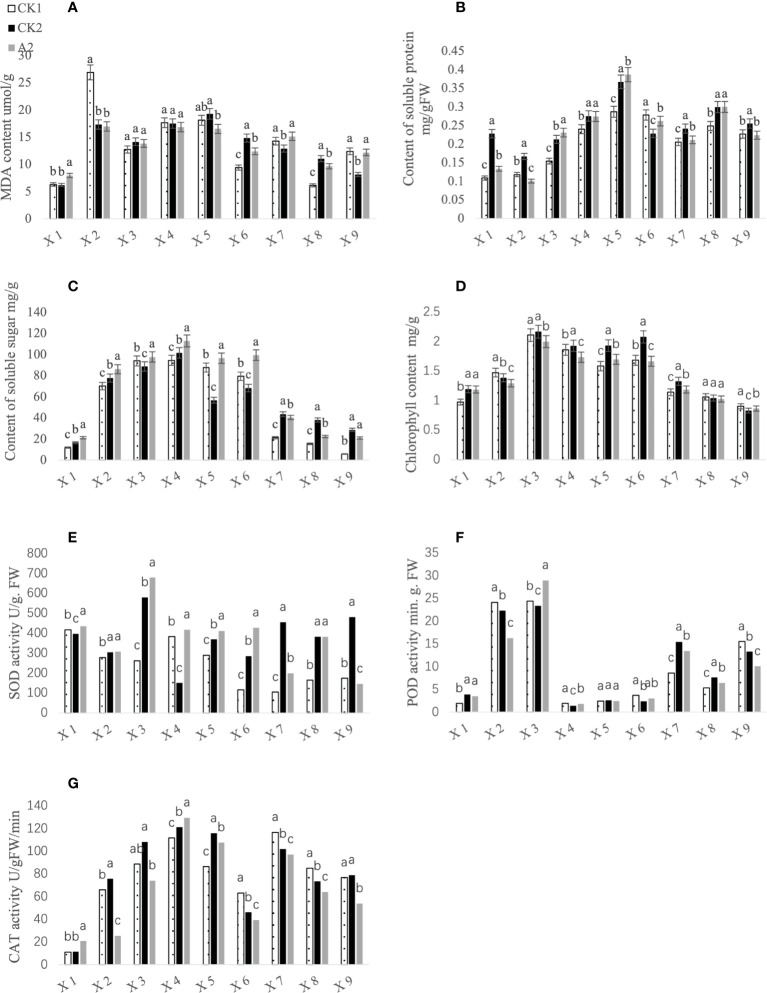
Effects of different treatments on physiological and biochemical indices of Fanming No. 1 leaves. X1–X9 represent the seedling period, 5–6-leaf period, bud stage, initial bloom stage, full bloom stage, final bloom stage, day 21 of pod growth, day 26 of pod growth, and day 31 of pod growth, respectively. **(A–G)** represents MDA content, Content of soluble protein, Content of soluble protein, Content of soluble sugar, Chlorophyll content, SOD activity, POD activity and CAT activity, respectively. Different letters indicate significant differences (*p* < 0.05).

The physiological and biochemical indicators of CK1 (blank control), CK2 (brassinolide treatment only), and A2 (brassinolide treatment followed by glufosinate treatment) leaves at different growth stages were measured. The results showed that the catalase activity (**Figure 2G**), SOD activity (**Figure 2E**), soluble sugar content (**Figure 2C**), and malondialdehyde content (**Figure 2A**) after A2 treatment were significantly higher than those of the control at the seedling stage. After entering the 5–6 leaf period, the content of soluble substances in the A2 treatment group increased and was significantly higher than that in the control group. The contents of soluble sugar and soluble protein (**Figure 2B**) first increased and then decreased, and the content of soluble sugar in the A2 treatment group was highest in the initial bloom stage, whereas the highest soluble protein content was observed in the full bloom stage. Peroxidase activity (**Figure 2F**) and SOD activity in the A2 treatment group were highest at the bud stage and were significantly higher than those in the control group. The chlorophyll content (**Figure 2D**) was higher in the CK2 treatment group than in the other treatments, except for at the 5–6 leaf periods and at days 26 and 31 of pod growth.

#### 3.2.2 Analysis of agronomic character index of Fanming No. 1

The results of the prewinter survey are shown in **Table 3**. Except for the largest leaf length, the growth of Fanming No. 1 under A2 treatment was poor, followed by CK2 and CK1 treatments. These results indicate that brassinolide treatment alleviated the effects of glufosinate. **Table 4** shows the growth status of Fanming No. 1 at the maturity stage of different treatments, CK2 outperforms both CK1 and A2 treatments in terms of plant height (cm), primary effective branch site (cm), effective length of the main inflorescence (cm), number of effective branches at a time, number of effective pods on the main inflorescence, and grain weight per plant (g). The number of effective pods in the whole plant of the A2 treatment group was higher than that in the CK1 and CK2 groups, and the pod length and seed number per plant of the CK1 treatment pods were larger than those in the CK2 and A2 groups.

**Table 3:** The growth status of rapeseed seedlings under different treatments before winter.

Materials	Green leaves of main stem	Total leaves of main stem	The largest leaf		Width of rootstock (cm)	Plant height (cm)
			Length (cm)	Width (cm)		
CK1	10.8 ± 1.92^a^	12.8 ± 1.30^a^	27.8 ± 2.51^a^	19.5 ± 2.72^a^	1.994 ± 0.40^a^	22.2 ± 2.17^a^
CK2	9.6 ± 1.52^a^	11.4 ± 1.52^ab^	26.5 ± 3.34^a^	19.1 ± 2.58^a^	1.74 ± 0.39^ab^	19.6 ± 3.13^b^
A2	9.4 ± 1.14^a^	10.8 ± 1.10^b^	28.62 ± 1.08^a^	19.06 ± 0.86^a^	1.38 ± 0.08^b^	17.72 ± 3.20^b^

CK1, blank control, CK2 brassinolide treatment only; A2, brassinolide treatment followed by glufosinate treatment. Different letters indicate significant differences (*p* < 0.05).

**Table 4:** Analysis of agronomic characters of Fanming No. 1 under different treatments.

Materials	Plant height (cm)	Primary effective branch site (cm)	Effective length of main inflorescence (cm)	Number of effective branches at a time	Number of effective pods on main inflorescence	Effective pods number of whole plant	Pod length (cm)	Seeds number of per pod	Grain weight of per plant (g)
CK1	177.4 ± 6.80^b^	86.4 ± 2.07^c^	36.0 ± 2.54^c^	9.8 ± 1.79^c^	48.0 ± 6.04^b^	328.4 ± 8.53^c^	6.22 ± 0.36^a^	23.88 ± 1.53^a^	29.3 ± 0.66^c^
CK2	184.0 ± 3.32^a^	122.2 ± 5.50^a^	60.0 ± 2.83^a^	12.4 ± 1.52^a^	71.6 ± 8.44^a^	434 ± 29.89^a^	5.88 ± 0.44^a^	21.6 ± 4.93^a^	31.3 ± 1.09^a^
A2	172.0 ± 3.08^b^	105.4 ± 2.41^b^	46.8 ± 2.17^b^	11.8 ± 0.84^b^	48.0 ± 2.55^b^	500 ± 17.14^b^	5.89 ± 0.36^a^	22.8 ± 3.15^a^	31.0 ± 1.54^b^

CK1, blank control, CK2 brassinolide treatment only; A2, brassinolide treatment followed by glufosinate treatment. Different letters indicate significant differences (*p* < 0.05).

#### 3.2.3 Analysis of disease resistance of Fanming No. 1

The disease survey results; disease index. As shown in **Table 5**, CK2 had the lowest disease index (<7.5) and incidence (<15%), followed by A2, whereas CK1 had the highest. These results indicate that CK2 had stronger resistance to sclerotinia and that brassinolide improves the disease resistance of Fanming No. 1.

**Table 5:** Disease survey results.

Sample	Grading of disease/plant							
	Lever 0	Lever 1	Lever 2	Lever 3	Lever 4	Total/plant	Disease index	Morbidity/%
CK 1	15	2	1	1	1	20	13.75	25%
CK 2	17	1	1	1	0	20	7.5	15%
A 2	16	2	0	1	1	20	11.25	20%

CK1, blank control, CK2 brassinolide treatment only; A2, brassinolide treatment followed by glufosinate treatment.

#### 3.2.4 *ATG3* expression analysis

**Figure 3:**
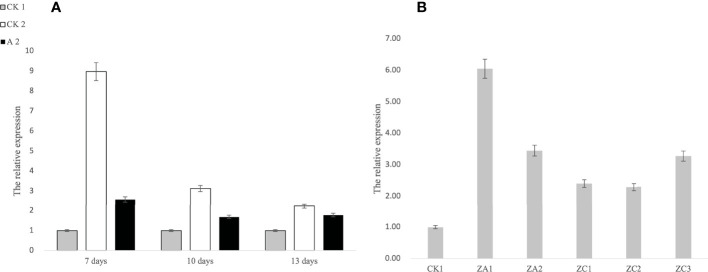
Expression levels of *ATG3* at different stages under different treatments in *Brassica napus.* **(A)** 1–3 correspond to 7, 10, and 13 days after herbicide treatment, respectively; **(B)** CK1 is the control, ZA1 and ZA2 are the *ALDH3F1* transgenic rape specimens, and ZC1–ZC3 are the *CYP90A1* transgenic rape specimens.

The expression level of *ATG3* in Fanming No. 1 different treatment groups and at different periods was measured. As shown in **Figure 3A**, both CK2 and A2 treatments showed upregulated ATG3 expression 7, 10, and 13 days after herbicide treatment, with the highest *ATG3* expression observed after CK 2 and A2 treatment in the first period. These results indicate that brassinolide treatment improved the resistance of Fanming No. 1 to sclerotinia disease. Furthermore, in transgenic seedlings overexpressing *ALDH3F1* and *CYP90A1*, the expression of *ATG3* was 3.39–6.11- and 2.29–3.13-fold higher than that in the control (**Figure 3B**), respectively.

### 3.3 Differential gene screening

#### 3.3.1 Transcriptome analysis of seedlings

##### 3.3.1.1 Sequencing quality statistics

The leaves of glufosinate-treated Fanming No. 1 plants on day 7 after treatment and leaves of control plants were collected to extract RNA and evaluate RNA quality. The RNA integrity number values of extracted RNA after each treatment were higher, with an OD260/280 and OD260/230 >2. The samples were sequenced and the sequencing data were further filtered. The contents of Q20, Q30, clean reads, and clean data were >90% (**Table 6**), indicating that the sequencing results were reliable. The upgraded version of HISAT2 (http://ccb.jhu.edu/software/hisat2/index.shtml) software of TopHat2 was used to align the filtered reads to the reference genome; >90% of the total reads were mapped (**Table 6**), indicating that the reference genome was selected appropriately and was free of contamination.

**Table 6:** Assessment of library quality and analysis of comparison results.

Sample	Reads no.	Q20 (%)	Q30 (%)	Clean reads no.	Clean data (%)	Total mapped (%)	Uniquely mapped (%)	Mapped to gene (%)	Mapped to exon (%)
C1	49408952	97.46	92.72	45079408	91.23	41583542 (92.25%)	39534424 (95.07%)	36651293 (92.71%)	36135281 (98.59%)
C2	46986734	97.96	94.23	43036380	91.59	39781094 (92.44%)	37880284 (95.22%)	35044120 (92.51%)	34513797 (98.49%)
C3	47377334	98	94.25	43534556	91.88	40298130 (92.57%)	38594626 (95.77%)	35802265 (92.76%)	35272204 (98.52%)
CK1	45548330	97.46	93.14	41737090	91.63	38667067 (92.64%)	36646044 (94.77%)	34708273 (94.71%)	34316012 (98.87%)
CK2	48413974	97.49	93.18	44289076	91.47	41008648 (92.59%)	38809486 (94.64%)	36749688 (94.69%)	36342188 (98.89%)
CK3	46391096	97.5	93.25	42261758	91.09	39125218 (92.58%)	37065052 (94.73%)	35109083 (94.72%)	34722181 (98.90%)

CK1-CK3, blank control, C1-C3 is brassinolide treatment followed by glufosinate treatment.

##### 3.3.1.2 Analysis of expression differences

**Figure 4:**
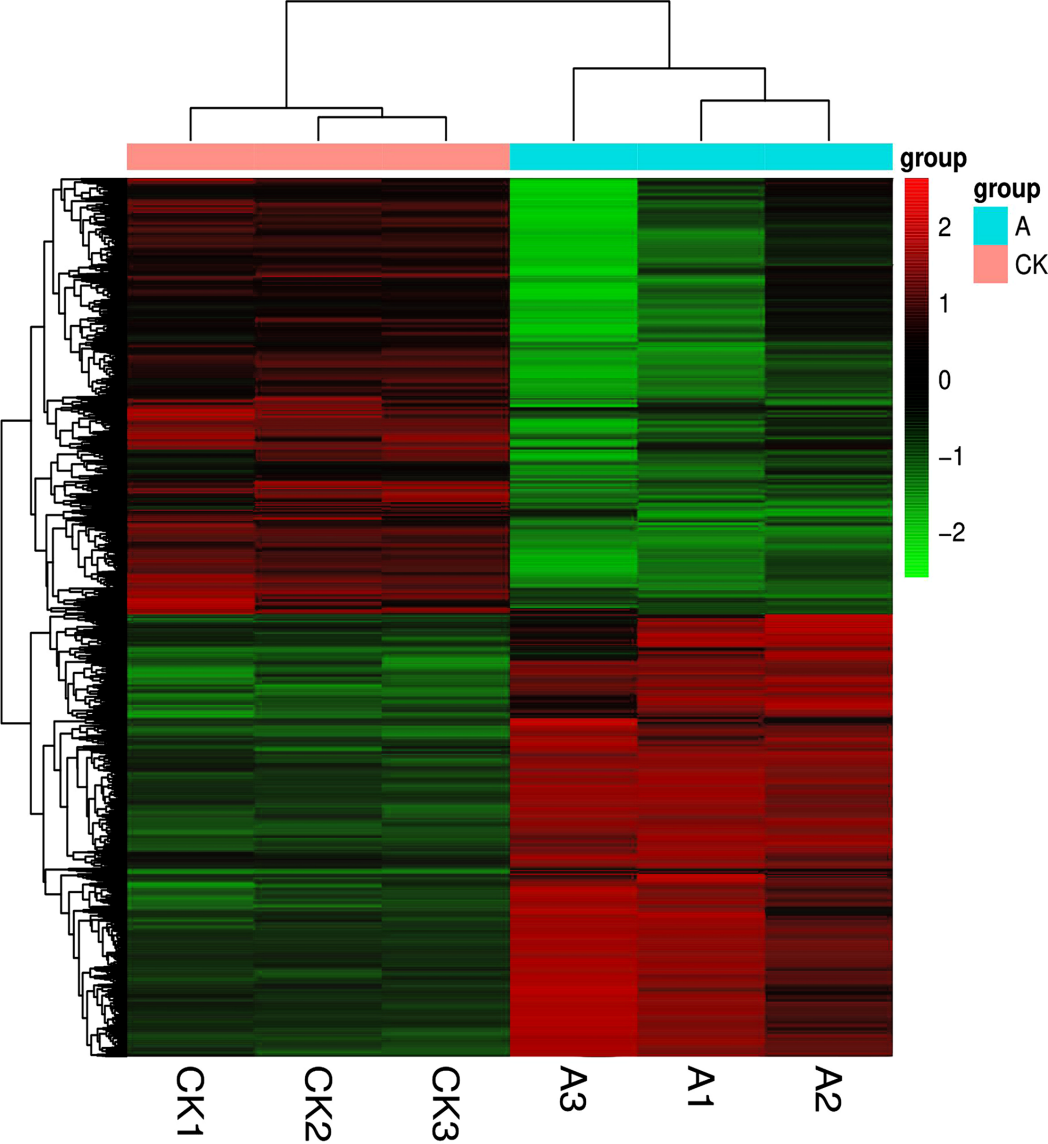
Clustering of differentially expressed genes. The horizontal direction represents genes; each column is a sample; red indicates highly expressed genes, and green indicates low gene expression.

The R language Pheatmap software package was used to perform bidirectional clustering analysis on the union for cluster and samples with differentially expressed genes (DEGs) in each group. The expression of the evaluated genes differed, as shown in **Figure 4**.

#### 3.3.2 Analysis of functional enrichment of DEGs

##### 3.3.2.1 Gene Ontology enrichment analysis

**Figure 5:**
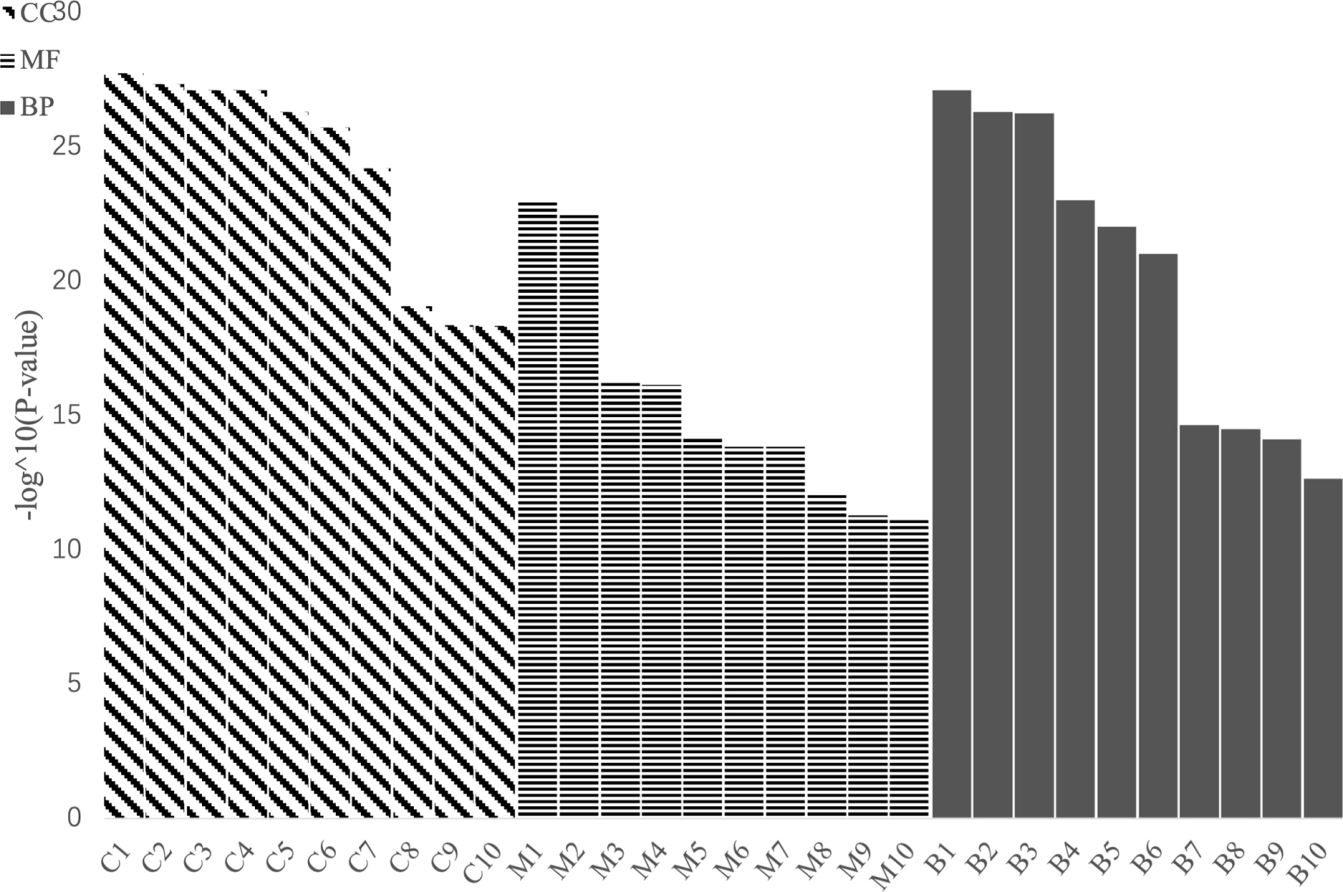
Histogram of Gene Ontology (GO) enrichment analysis (CC, cell components; MF, molecular functions; BP, biological processes). C1–C10 represent GO:0034357 photosynthetic membrane, GO:0009521 photosystem, GO:0009579 thylakoid, GO:0044436 thylakoid part, GO:0009523 photosystem II, GO:0042651 thylakoid membrane, GO:0009654 photosystem II evolving oxygen complex, GO:0005840 ribosome, GO:1990204 oxidoreductase complex and GO:0030529 intracellular ribonucleoprotein complex; M1–M10 respectively GO:0005198 structural molecule activity, GO:0003735 structural constituent of ribosome, GO:0003824 catalytic activity, GO:0036094 small molecule binding, GO:0004713 protein tyrosine kinase activity, GO:0000166 nucleotide binding, GO:1901265 nucleoside phosphate binding, GO:0043168 anion binding, GO:0004674 protein serine/threonine kinase activity and GO:0016740 transferase activity; B1–B10 represent GO:0015979 photosynthesis, GO:0044249 cellular biosynthetic process, GO:1901576 organic substance biosynthetic process, GO:0009058 biosynthetic process, GO:0044237 cellular metabolic process, GO:1901566 organonitrogen compound biosynthetic process, GO:0009987 cellular process, GO:0044271 cellular nitrogen compound biosynthetic process, GO:0008152 metabolic process, GO:0043043 peptide biosynthetic process.

Gene Ontology (GO) enrichment analysis showed that the DEGs were mainly enriched in metabolic pathways, such as the photosynthetic membrane (135 DEGs), photosystem (122 DEGs), thylakoid (137 DEGs), thylakoid part (137 DEGs), photosynthesis (146 DEGs), and cellular biosynthetic processe (1692 DEGs) (**Figure 5**). Glufosinate mainly affected the photosynthesis and cell growth of *B. napus*.

##### 3.3.2.2 Kyoto Encyclopedia of Genes and Genomes enrichment analysis

According to Kyoto Encyclopedia of Genes and Genomes enrichment analysis of the DEGs, 121 metabolic pathways showed differences, among which 40 showed the *p*-value below a specific threshold and false discovery rate <0.3. These pathways were mainly divided into lipid metabolism (9), amino acid metabolism (8), carbohydrate metabolism (5), cofactor and vitamin metabolism (4), other secondary metabolite synthesis (2), signaling (2), nucleotide metabolism (2), folding (2), transport and catabolism (2), energy metabolism (1), other amino acid metabolism (1), terpenoid and polyketide metabolism (1), and glycan biosynthesis and metabolism (1) (**Table 7**). Amino acid metabolism, secondary metabolite synthesis, energy metabolism, carbohydrate metabolism, lipid metabolism, other amino acid metabolism, and metabolism of terpenoids and polyketides are closely related to growth and development, indicating that soaking of Fanming No. 1 seedlings in brassinolide mainly affected growth and development.

**Table 7:** Kyoto Encyclopedia of Genes and Genomes enrichment pathway analysis of DEGs.

Pathway ID	Pathway	Up number	Down number	Level
*bna00500*	Starch and sucrose metabolism	59	111	Carbohydrate metabolism
*bna00010*	Glycolysis/gluconeogenesis	45	113	
*bna00053*	Ascorbate and aldarate metabolism	15	46	
*bna00620*	Pyruvate metabolism	32	85	
*bna00520*	Amino sugar and nucleotide sugar metabolism	58	109	
*bna00910*	Nitrogen metabolism	24	36	Energy metabolism
*bna00260*	Glycine, serine and threonine metabolism	36	87	Amino acid metabolism
*bna00350*	Tyrosine metabolism	28	29	
*bna00220*	Arginine biosynthesis	19	40	
*bna00360*	Phenylalanine metabolism	42	26	
*bna00250*	Alanine, aspartate and glutamate metabolism	25	57	
*bna00270*	Cysteine and methionine metabolism	62	98	
*bna00340*	Histidine metabolism	8	30	
*bna00330*	Arginine and proline metabolism	49	33	
*bna00073*	Cutin, suberine and wax biosynthesis	4	46	Lipid metabolism
*bna00591*	Linoleic acid metabolism	9	9	
*bna00564*	Glycerophospholipid metabolism	61	60	
*bna00062*	Fatty acid elongation	9	39	
*bna00592*	alpha-Linolenic acid metabolism	41	18	
*bna00561*	Glycerolipid metabolism	44	59	
*bna00565*	Ether lipid metabolism	21	16	
*bna00071*	Fatty acid degradation	36	28	
*bna00600*	Sphingolipid metabolism	24	12	
*bna00960*	Tropane, piperidine and pyridine alkaloid biosynthesis	23	32	Biosynthesis of other secondary metabolites
*bna00945*	Stilbenoid, diarylheptanoid and gingerol biosynthesis	18	2	
*bna00906*	Carotenoid biosynthesis	22	34	Metabolism of terpenoids and polyketides
*bna00670*	One carbon pool by folate	3	32	Metabolism of cofactors and vitamins
*bna00785*	Lipoic acid metabolism	0	11	
*bna00780*	Biotin metabolism	7	24	
*bna00740*	Riboflavin metabolism	3	18	
*bna00450*	Selenocompound metabolism	11	18	Metabolism of other amino acids
*bna04146*	Peroxisome	49	78	Transport and catabolism
*bna04136*	Autophagy – other	54	4	
*bna04016*	MAPK signaling pathway-plant	124	55	Signal transduction
*bna04075*	Plant hormone signal transduction	256	131	
*bna00240*	Pyrimidine metabolism	29	60	Nucleotide metabolism
*bna00230*	Purine metabolism	45	90	
*bna04141*	Protein processing in endoplasmic reticulum	124	152	Folding, sorting and degradation
*bna03060*	Protein export	16	62	
*bna00514*	Other types of O-glycan biosynthesis	2	3	Glycan biosynthesis and metabolism

#### 3.3.3 Glufosinate-related metabolic pathway analysis

We performed GO and Kyoto Encyclopedia of Genes and Genomes pathway enrichment analyses, focusing on DEGs in herbicide-related pathways, including photosynthesis (Song et al., 2020), pyruvate metabolism (Leslie and Baucom, 2014), and amino acid metabolism (Zhao, N. et al., 2017).

##### 3.3.3.1 Photosynthesis

**Figure 6:**
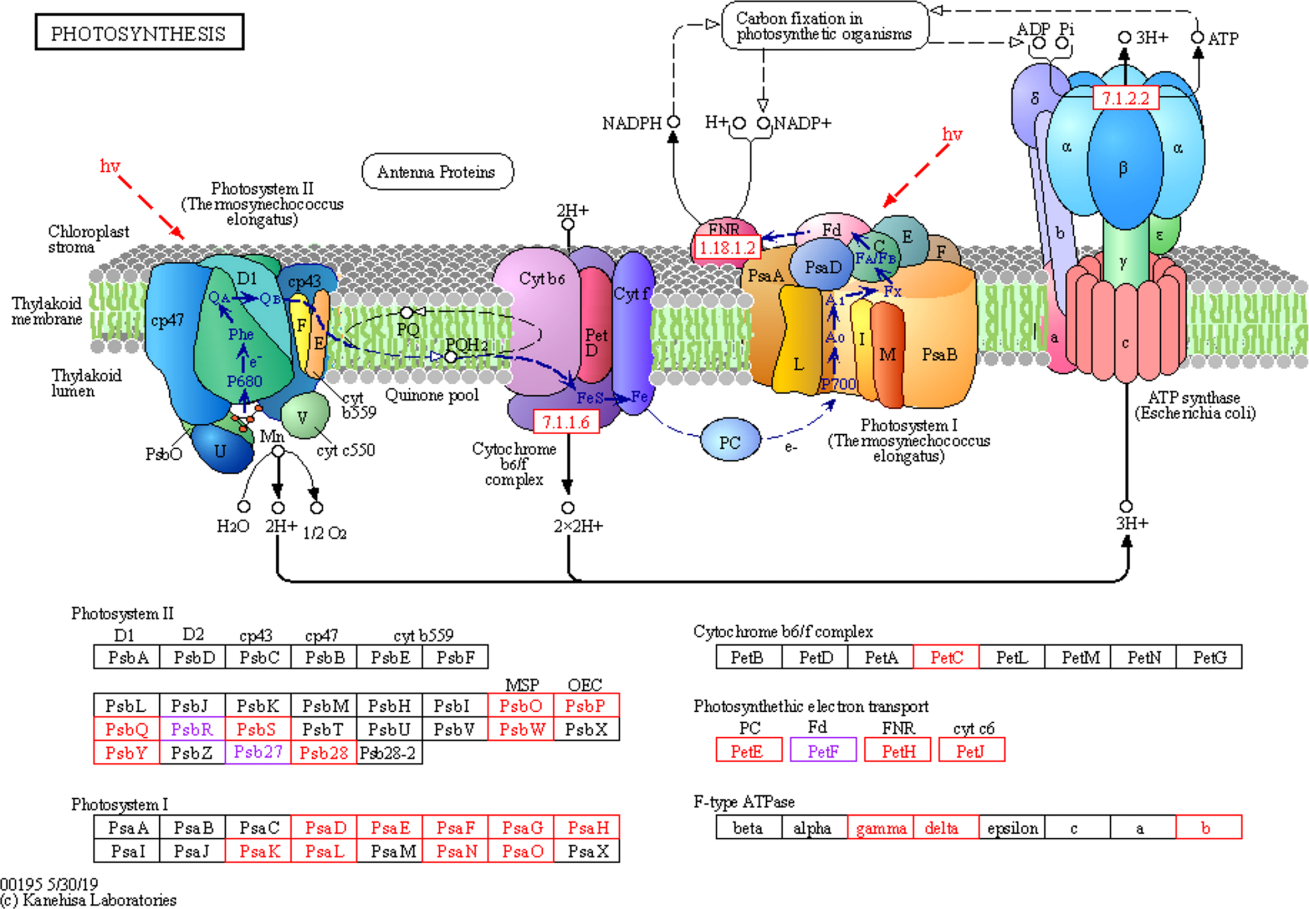
Photosynthesis metabolic pathways involving differentially expressed genes. Photosystem II, photosystem I, cytochrome b6/f complex, photosynthetic electron transport, F-type ATPase, carbon fixation in photosynthetic organisms, chloroplast stroma, thylakoid membrane, and thylakoid lumen.

After spraying with glufosinate, nine DEGs were identified in photosystem I, all of which were downregulated. Nine DEGs were identified in photosystem II, of which two genes were upregulated. Seven genes were downregulated in photosystem II. One DEG in the cytochrome b6/f complex was downregulated. There were four DEGs involved in photosynthetic electron transport, with one node gene that was upregulated and three genes that were downregulated. Three F-type ATPases were downregulated (**Figure 6**).

##### 3.3.3.2 Pyruvate metabolism

EPSP synthase is an enzyme involved in synthesizing aromatic amino acids by catalyzing the reaction of shikimate-3-phosphate and phosphoenolpyruvate to synthesize 5-enolpyruvylshikimate-3-phosphate. After spraying with pesticides, 114 genes in the pyruvate metabolic pathway showed differences in expression levels. Among them, three gene nodes were upregulated, five gene nodes were downregulated, and ten gene nodes were both upregulated and downregulated. Most of these genes are related to EPSP synthase, indicating that glufosinate affects the biosynthesis of pyruvate and leads to changes in EPSP synthase.

##### 3.3.3.3 Amino acid metabolism

After spraying with glufosinate, 669 genes were differentially expressed in amino acid metabolism pathways, including in arginine biosynthesis; tyrosine metabolism; and glycine, serine, and threonine metabolism, of which 269 genes were downregulated and 400 genes were upregulated.

### 3.4 qRT-PCR analysis of glufosinate resistance-related genes

**Figure 7:**
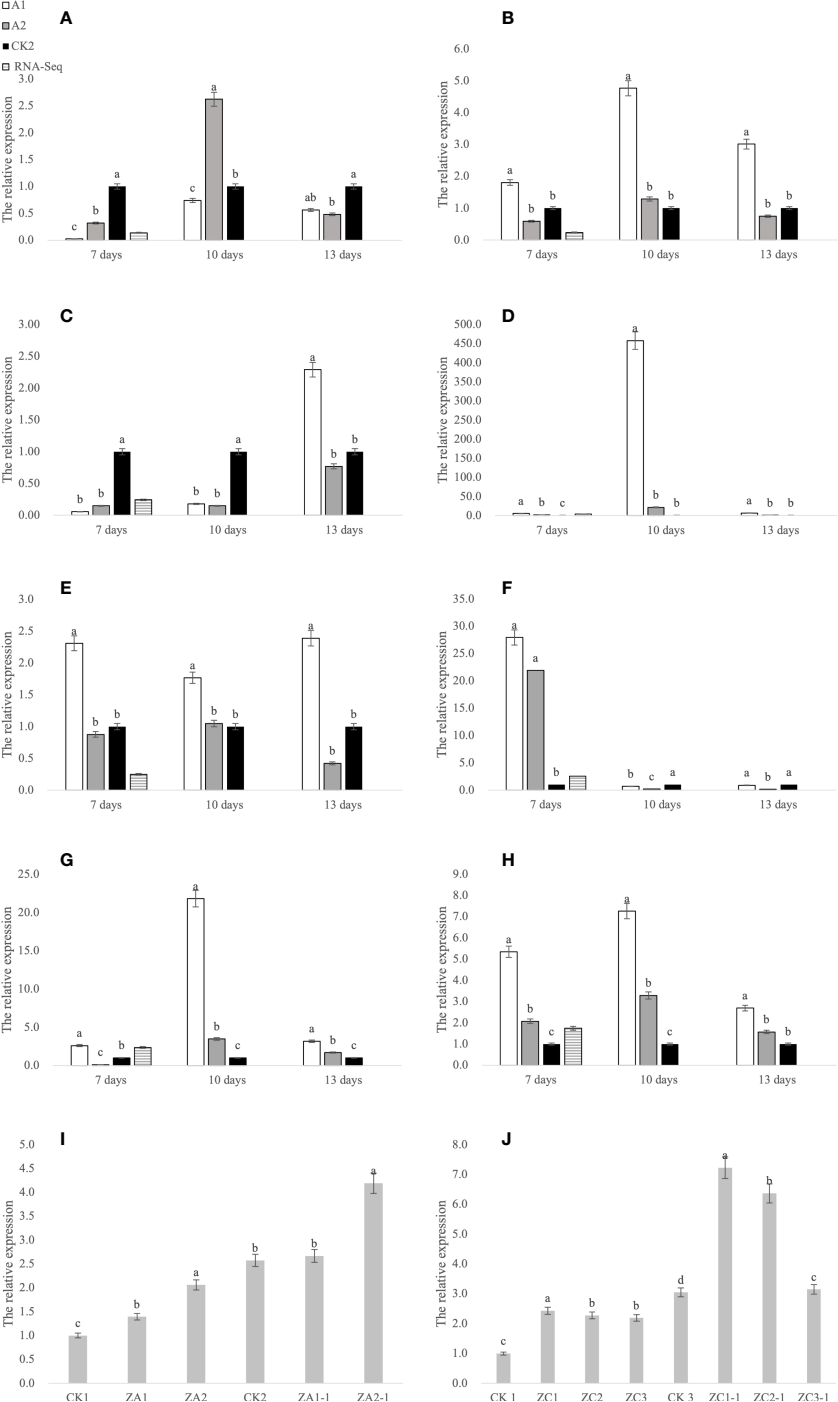
qRT-PCR analysis of genes. **(A–H)** Gene expression at 7, 10, and 13 days after glufosinate treatment. The relative expression levels of A1 and A2 in the same period were divided by the relative expression levels of CK2, and CK2 was set to 1. **(I, J)** CK1 is the blank control; CK2 and CK3 are the expression levels of *ALDH3F1* and *CYP90A1* at 7 days after non-transgenic *Brassica napus* seedlings were treated with glufosinate-ammonium, respectively; ZA1 and ZA2 are the expression levels of *ALDH3F1* in the transgenic *ALDH3F1* seedlings; ZC1–ZC3 denote the expression level of *CYP90A1* in *CYP90A1* transgenic seedlings; and ZA1-1, ZA2-1, and ZC1-1–ZC3-1 denote the expression levels of corresponding genes in transgenic seedlings treated with glufosinate. Different letters indicate significant differences (p < 0.05).

According to the sequencing results, numerous genes affected glufosinate resistance in Fanming No. 1, including *LHCB1* (*BnaA05g09410D*, **Figure 7A**), *fabF* (*BnaA06g36060D*, **Figure 7B**), *psbW* (*BnaA04g17660D*, **Figure 7C**), *CYP90A1* (*BnaA10g24860D*, **Figure 7D**), *ALDH3F1* (*BnaA03g59170D*, **Figure 7E**), *ACOX1* (*BnaC08g23150D*, **Figure 7F**), *petF* (*BnaA03g22350D*, **Figure 7G**), and *ACSL* (*BnaC01g15670D*, **Figure 7H**). We collected samples from the CK1 (**Figure 1A**) and CK2 (**Figure 1B**), glufosinate-only treatment (death on day 7; **Figure 1E**), A1 (glyphosate-only treatment, death on day 15, **Figure 1D**), A2 (survival, **Figure 1C**), 7 and 10 days after glufosinate treatment, and 13-day groups to determine the expression levels of each gene at different stages.

This eight key glufosinate resistance genes were evaluated using qRT-PCR. **Figure 7** shows the expression levels of these genes at 7 days after glufosinate treatment. The first period of each graph revealed changes in the expression level of each gene according to qRT-PCR (A2) and RNA-Seq. **Figure 6** shows that the trends in the expression levels determined in qRT-PCR and RNA-Seq were highly similar, indicating that the expression data obtained using RNA-Seq are reliable.

### 3.5 Functional validation of two key differential genes

#### *3.5.1 Agrobacterium*-mediated transformation of the hypocotyl of *B. napus*

**Figure 8:**
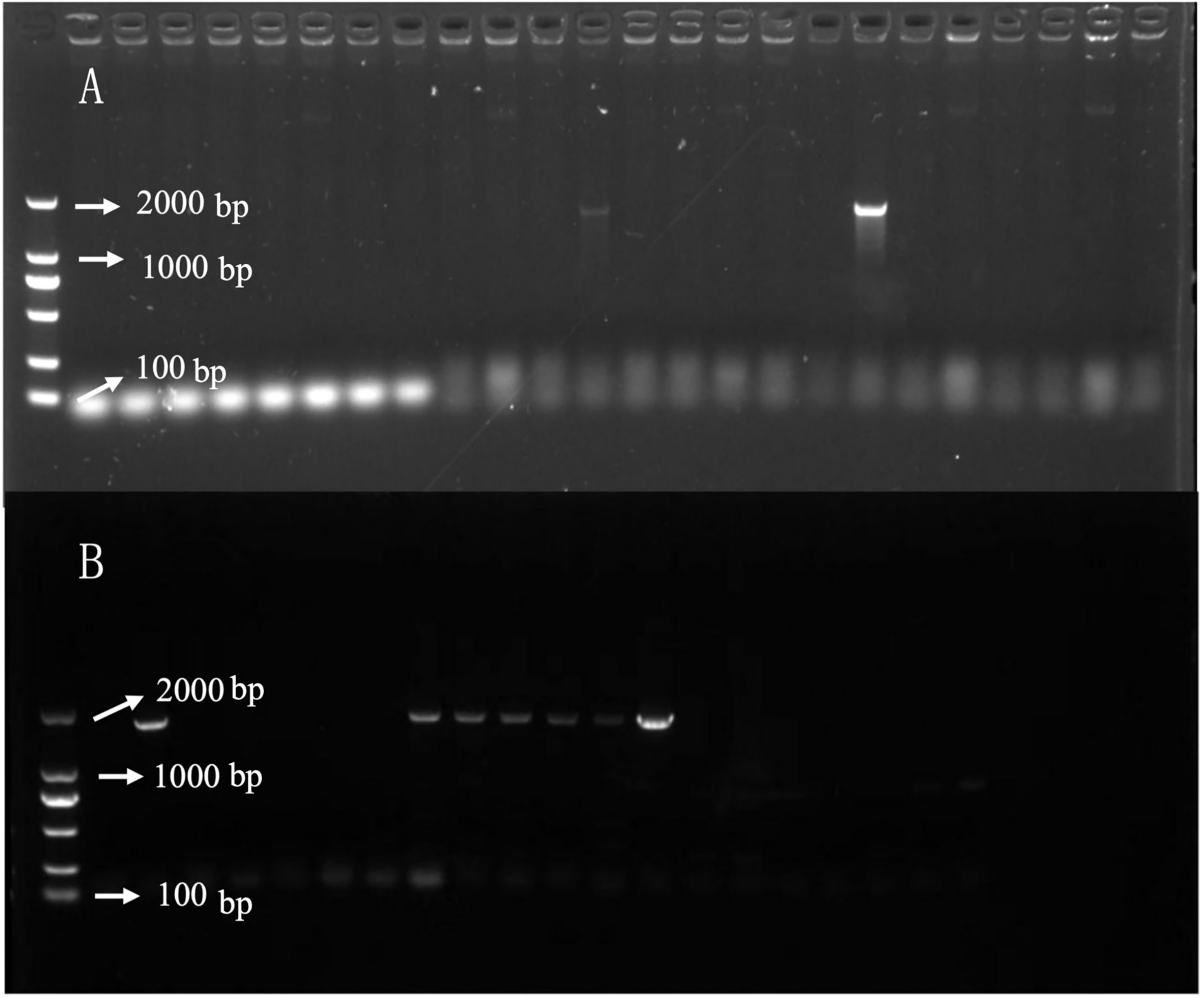
DNA detection of *ALDH3F1* and *CYP90A1* in transgenic plants. **(A, B)** are PCR detection of transgenic plants overexpression ALDH3F1 and CYP90A1 respectively.

Because *B. napus* died 13 days after treatment following A1 treatment and the specimen in the A2 group turned green 13 days after treatment, gene expression was compared in the two (herbicide) treatment groups. In gene-corresponding protein function analysis, two key genes were identified, *CYP90A1* and *ALDH3F1*. *Agrobacterium*-mediated hypocotyl transformation was performed to transform the recombinant expression vectors pBI121-CYP90A1 and pBI121-ALDH3F1 into *B. napus* Zhongshuang 11 to obtain transgenic *B. napus* plants. DNA was extracted from the transgenic plants for PCR verification and pBI121 was obtained: pBI121-ALDH3F1 (**Figure 8A**) and pBI121-CYP90A1 (**Figure 8B**). Next, the expression levels of *CYP90A1* and *ALDH3F1* in the PCR positive plants were evaluated using qRT-PCR. Under the action of strong promoters, the *CYP90A1* expression levels of positive plant ZC1, ZC2 and ZC3 increased by 1.43, 1.27- and 1.19-fold, respectively (**Figure 7J**). In the control, the *ALDH3F1* expression levels of positive plant ZA1 and ZA2 were 1.40- and 2.06-fold as high as those in the control, respectively (**Figure 7I**).

#### 3.5.2 Resistance identification of transgenic plants

After treatment of control and transgenic rape seedlings with glufosinate for 7 days, the leaves of plants in the blank control group showed yellowing and shrinkage. The leaves of *ALDH3F1* transgenic rape seedlings shrank significantly, although no yellowing was observed (**Figure 1G**). *CYP90A1* transgenic rape seedlings showed slight shrinkage without yellowing (**Figure 1H**). After 7 days of glufosinate treatment, samples were collected for qRT-PCR. In the overexpression lines ZA1-1 and ZA2-1, the relative expression levels of *ALDH3F1* were 1.04- and 1.63-fold as high as that in the control, respectively (**Figure 7I**). In the overexpression lines ZC1-1,ZC2-1 and ZC3-1,the *CYP90A1* expression was 2.37-, 2.09-, and 1.03-fold as high as that in the control (**Figure 7J**). The expression levels of *ALDH3F1* and *CYP90A1* were higher in the overexpression lines than in control plants.

## 4 Discussion

### 4.1 Key genes in response to glufosinate stress under brassinolide treatment

When plants are externally stimulated, their gene expression is altered to mitigate changes in the environment. We found that *B. napus* pre-treated with brassinolide developed resistance to sclerotinia and glufosinate. Following transcriptome analysis, the GO enrichment results were mainly concentrated in the photosynthetic membrane, photosystem, and photosynthesis. The Kyoto Encyclopedia of Genes and Genomes enrichment results were also concentrated in carbohydrate metabolism, energy metabolism, and amino acid metabolism, indicating that pre-treatment with brassinolide affected plant growth and development and alleviated glufosinate stress in Fanming No. 1.

During the plant stress response, we evaluated the expression of eight key glufosinate resistance genes. The expression levels of *CYP90A1* and *ALDH3F1* were significantly different compared to in the control, with the expression levels gradually decreasing as the plants recovered. These two genes were further analyzed.

### 4.2 Overexpression of *CYP90A1* and *ALDH3F1* contributes to resistance of *B. napus* to glufosinate

Aldehydes are intermediates in several fundamental metabolic pathways. Aldehyde dehydrogenase (ALDH) enzymes belong to a family of NAD(P)^+^-dependent enzymes, which exhibit substrate specificity and catalyze the oxidation of various aldehydes to the corresponding carboxylic acids, thereby reducing lipid peroxidation (Kirch et al., 2004). Plant family 2 ALDHs have been suggested to oxidize acetaldehyde generated *via* ethanolic fermentation, producing acetate for acetyl-CoA biosynthesis *via* acetyl-CoA synthetase, similar to the yeast pathway named as “pyruvate dehydrogenase bypass” (Wei et al., 2009). Transgenic *Arabidopsis* plants overexpressing *ALDH3F1* are more tolerant to salt (NaCl and/or KCl), dehydration, and oxidative stress (Stiti et al., 2011). Activation of NADPH oxidase results in higher levels of H_2_O_2_, which triggers the relevant sensor to stimulate the mitogen-activated protein kinase cascade in plants under Cd stress. Mitogen-activated protein kinase activates the function of *trans*-regulatory elements in the nucleus to bind *cis*-regulatory elements and subsequently enhance the transcriptional levels of SOD and catalase to alleviate plant stress (Planas-Riverola et al., 2019), which is consistent with our results. We found that *ALDH3F1* was highly expressed under the influence of glufosinate-ammonium stress; over time, the plants gradually recovered and *ALDH3F1* expression decreased. These results suggest that high *ALDH3F1* expression enhances the tolerance of Zhongshuang 11 plants to glufosinate.

The functions of cytochrome P450 monooxygenase *CYP90A1/CPD* (mutants identified in *Arabidopsis*) include constitutive photomorphogenesis and dwarfism (cpd; *CYP90A1* deficiency). The expression levels of *CYP90A1/CPD* are correlated with the spatial allocation of the *CYP90A1* substrate 6-deoxocatha-sterone, suggesting that *CYP90* genes contribute to the regulation of brassinolide biosynthesis (Bancoş et al., 2002). Most herbicides such as prosulfuron, diclofop and chlortoluron can be converted into several metabolites by P450 (Danièle et al., 2000). P450 primarily catalyzes the monooxygenation of lipophilic xenobiotics, including herbicides, and plays a major role in the oxidation of most classes of herbicides (Frear, 1995). We found that *CYP90A1* was highly expressed during glufosinate stress. Over time, the plants gradually recovered, and *CYP90A1* expression decreased. These results suggest that high *CYP90A1* expression enhances the tolerance of Zhongshuang 11 tolerance to glufosinate.

### 4.3 Overexpression of *CYP90A1* and *ALDH3F1* leads to upregulated *ATG3* expression

*ATG3* is crucial for responses to various biotic and abiotic stressors in animals and plants. A study of autophagy regulation in tomato showed that the heat shock transcription factor HsfA1a confers drought tolerance and induces autophagy by activating ATG (Wang et al., 2015). In this study, we found that the incidence of sclerotinia in samples treated with brassinolide and herbicides after brassinolide treatment was lower than that in the control, and the expression of *ATG3* was upregulated at all three time points evaluated. These results indicate that upregulation of *ATG3* expression can enhance the resistance of rape to sclerotiorum, which is consistent with the results of Wang et al. (2019).

*ATG3* was highly expressed in plants overexpressing *ALDH3F1* and *CYP90A1*. These results indicate that upregulated expression of *ALDH3F1* and *CYP90A1* leads to upregulated expression of *ATG3*, which may cause Zhongshuang 11 to be resistant to sclerotiorum. The incidence of sclerotinia in later stages will be verified in follow-up experiments.

## 5 Conclusion

Soaking of *B. napus* seeds in 0.015% brassinolide for 6 h resulted in the germination rate. Brassinolide treated plants started to turn green and continued to grow on day 7 of glufosinate application, indicating that the treatment conferred some resistance to glufosinate. The incidence of sclerotinia disease was reduced by 10% and the disease index was reduced by 6 points in the brassinolide-pre-treated group compared to in the control. Additionally, the activities of catalase and SOD increased under brassinolide pre-treatment, increasing the content of soluble substances. Transgenic seedlings overexpressing *ALDH3F1* shrunk but did not show yellowing, whereas seedlings overexpressing *CYP90A1* showed only slight shrinkage, suggesting that transgenic Zhongshuang No. 11 was strongly resistant to glufosinate. Moreover, the expression level of related genes in transgenic plants and expression level of the antibacterial *S. sclerotiorum* gene *ATG3* in transgenic plants were higher than those in the control. These results indicate that *ALDH3F1* and *CYP90A1* are improve the resistance of *B. napus* to sclerotium disease. Our results provide a theoretical basis for the molecular breeding of *B. napus* to improve disease and glufosinate resistance, thereby promoting the development of the rapeseed industry.

## Data availability statement

The datasets presented in this study can be found in online repositories. The names of the repository/repositories and accession number(s) can be found below: https://www.ncbi.nlm.nih.gov/, PRJNA877382.

## Author contributions

ZZ developed the experimental plan. ZL designed the experiments. QG conducted the experiments. ML wrote the manuscript. MH analyzed the data. All authors have read and approved the manuscript.

## Funding

This study was supported by the Natural Science Foundation of Changsha (kq2007015), National Transgenic Research Projects of China (2018ZX08020001), Agricultural Science, Technology Innovation Program (ASTIP) of CAAS (Grant No.2021IBFC), and Natural Science Foundation of China (31201240).

## Conflict of interest

The authors declare that this research was conducted in the absence of any commercial or financial relationships that could be construed as potential conflicts of interest.

## Publisher’s note

All claims expressed in this article are solely those of the authors and do not necessarily represent those of their affiliated organizations, or those of the publisher, the editors and the reviewers. Any product that may be evaluated in this article, or claim that may be made by its manufacturer, is not guaranteed or endorsed by the publisher.
